# Sunshine Policies and Murky Shadows in Europe: Disclosure of Pharmaceutical Industry Payments to Health Professionals in Nine European Countries

**DOI:** 10.15171/ijhpm.2018.20

**Published:** 2018-03-14

**Authors:** Alice Fabbri, Ancel.la Santos, Signe Mezinska, Shai Mulinari, Barbara Mintzes

**Affiliations:** ^1^Charles Perkins Centre and Faculty of Pharmacy, The University of Sydney, Camperdown, NSW, Australia.; ^2^Health Action International, Amsterdam, The Netherlands.; ^3^Faculty of Medicine and Institute of Clinical and Preventive Medicine, University of Latvia, Riga, Latvia.; ^4^Department of Sociology, Faculty of Social Sciences, Lund University, Lund, Sweden.

**Keywords:** Transparency, Pharmaceutical Industry, Conflict of Interest, Industry Relationships, Disclosure

## Abstract

Relationships between health professionals and pharmaceutical manufacturers can unduly influence clinical practice. These relationships are the focus of global transparency efforts, including in Europe. We conducted a descriptive content analysis of the transparency provisions implemented by February 2017 in nine European Union (EU) countries concerning payments to health professionals, with duplicate independent coding of all data. Using an author-generated, semi-structured questionnaire, we collected information from each disclosure policy/code on: target industries, categories of healthcare professionals covered, scope of payments included, location and searchability of the disclosed data. Our analysis shows that although important improvements have been put in place in the past few years, significant gaps remain in disclosure requirements and their implementation. The situation differs substantially from country to country and the most striking differences are between governmental and self-regulatory approaches, especially with regard to the comprehensiveness of the disclosed data. In many cases, individuals can still opt out and reporting is incomplete, with common influential gifts such as food and drink excluded. Finally, in several countries data are only available as separate PDFs from companies, thus making the payment reports difficult to access and analyse. In order to overcome these gaps, minimum standards for disclosures should be implemented across Europe. All payments to healthcare professionals and organizations should be included, all health-related industries should be required to submit reports, and usability of disclosed data should be guaranteed.

## Background

Financial ties between health professionals and pharmaceutical manufacturers have long been at the centre of international debate. Pharmaceutical companies invest large sums of money to interact with health professionals; in 2013, 20 drug companies spent a total of $14.8 billion in promotion, including traditional detailing, journal advertising, e-promotion, and professional meetings.^1^ Studies suggest that such relationships influence clinical practice and are associated with inappropriate and lower quality prescribing that can lead to negative effects on patient care and higher healthcare costs.^2,3^

These concerns have led to a range of national policies aimed to ensure greater disclosure of payments to health professionals by pharmaceutical manufacturers. For example, in 2013 the United States implemented the “Physician Payments Sunshine Act,” legislation passed in 2010 that requires drug and device companies to declare payments and hospitality to medical doctors and teaching hospitals.^4^ In Europe, the situation is still heterogeneous, with only some countries adopting legislation governing disclosure. A turning point has been the adoption in June 2013 of a Disclosure Code by the European Federation of Pharmaceutical Industries and Associations (EFPIA), the trade association of the research-based pharmaceutical industry. Since 2013, all 33 national industry associations that are EFPIA members – including five not European Union (EU) member states – have been mandated to implement disclosure programs.^5^ Disclosure rules must follow EFPIA guidelines, but exceptions are allowed when these provisions are in conflict with national laws or regulations.

The aim of this study is therefore to examine rules covering disclosure by pharmaceutical companies of their payments to health professionals in different European countries, in terms of comprehensiveness and ease of data access. We also make recommendations for the types of minimum standards needed for more comprehensive reporting standards.

## Methods

We conducted a descriptive content analysis of the national transparency provisions implemented by February 2017 in nine European countries and the provisions in the EFPIA code concerning payments to health professionals, with duplicate independent coding of all data, and any discrepancies resolved by consensus. If consensus could not be reached, coders consulted the study authors with a translation of the relevant text. This group of nine countries includes four countries from Western Europe (France, Germany, the Netherlands, the United Kingdom), one from Eastern Europe (Latvia), one from Northern Europe (Sweden), and three from Southern Europe (Italy, Portugal, Spain).

This convenience sample was chosen for regional variation, a mix of legislated and self-regulatory approaches, and availability of native-language data collectors with background knowledge of pharmaceutical policy. A call for data collectors was circulated among members of Health Action International, a non-governmental organisation representing the public interest in pharmaceutical policy, and the International Society of Drug Bulletins, a network of independent journals on therapeutics. In order to identify the codes/policies, each coder was provided with a paper that provides a brief overview of transparency regulations implemented in European countries.^6^ As the paper was published in 2014, coders were asked to conduct additional searches to identify new initiatives adopted since then. The focus of these searches was on government-led rules on payments disclosure (ie, in legislation/regulation) and self-regulatory initiatives (ie, in ethics codes of national pharmaceutical industry associations).

We designed a semi-structured questionnaire based on variables of theoretical interest based on the literature on industry-professional interactions and on previous analyses of transparency provisions.^6-8^ The questionnaire was pilot-tested within the research team.

From each policy/code, we collected the following information (see Supplementary file 1):

- general information (name of the code, organisation responsible, date of adoption);
- type of policy (governmental or self-regulatory) and applicability;
- target industries (eg, pharmaceutical, medical device industries, other health-related industries);
- categories of healthcare organisations and professionals covered (eg, medical doctors, pharmacists, nurses);
- provisions for reporting of individual data (eg, whether the consent of the recipient was required for publishing payments to named individual health professionals);
- scope of payments covered and presence of financial thresholds for reporting;
- location, searchability and user-friendliness of the data (user-friendliness was defined as the data collectors’ judgement on the format and usability of the data);
- sanctions for non-disclosure and monitoring of compliance.

We then conducted a descriptive data analysis, with a focus on differences between government and industry self-regulation, and on their strengths and limitations.

## Results

The Table provides an overview of the included policies. Additional information is available in Supplementary file 2. The focus of our analysis is primarily on rules covering disclosure by pharmaceutical companies of their payments to health professionals, but as shown in the Table, the codes also include requirements on disclosure of payments to healthcare organisations.

**Table:** Characteristics of the Transparency Provisions in Different EU Member States

	**DE**	**ES**	**FR**	**IT**	**LV** ^a^		**NL**	**SE**	**UK**	**PT**	**EFPIA** ^b^
Type of policy											
Industry self-regulation	Y	Y		Y		Y	Y^c^	Y	Y		Y
Government			Y		Y					Y	
Target industries											
Pharmaceutical industry	Y	Y	Y	Y	Y	Y	Y	Y	Y	Y	Y
Medical device industries			Y				Y^d^			Y	
Health professionals covered											
Physicians	Y	Y	Y	Y	Y	Y	Y	Y	Y	Y	Y
Non-physicians	Y	Y	Y	Y	Y	Y	Y	Y	Y	Y	Y
Organisations covered (eg, hospitals, medical centres, Universities, medical societies)											
	Y	Y	Y	Y	Y^e^	Y	Y^f^	Y	Y	Y	Y
Individual data
Mandatory data release		Y^g^	Y		Y		Y			Y	
Consent required	Y			Y		Y		Y	Y		Y
Threshold for disclosure											
	N	N	Y €10	N	N	N	Y €500/year per company	N	N	Y €60	N
Types of payments excluded from disclosure
Meals and drinks	Y	Y		Y		Y	Y	Y	Y		Y
Drug samples	Y	Y		Y	Y^h^	Y	Y	Y	Y	Y	Y
Transfers of value related to OTC drugs	Y	Y		Y		Y			Y		Y
Small gifts, education or promotional materials	Y	Y		Y		Y	Y	Y	Y		Y
Other					#		¨	*			
Location of the data and searchability											
Centralised searchable registry									Y		N/A^i^
Centralised searchable registry with no possibility for data extraction			Y^j^				Y^j^			Y	
Separate PDFs or weblinks on a single website					Y	Y		Y			N/A
Separate PDFs on each company website	Y	Y		Y		Y					N/A
Data access judged to be user-friendly											
	N	N	N	N	N	N	Partial	N	Y	Partial	N/A

Abbreviations: DE, Germany; ES, Spain; FR, France; IT, Italy; LV, Latvia; NL, The Netherlands; SE, Sweden; UK, United Kingdom; PT, Portugal; EFPIA, European Federation of Pharmaceutical Industries and Associations; EU, European Union; OTC, over-the-counter. ^a^ In Latvia governmental and industry regulation coexists, with complementary roles. ^b^ The EFPIA Code is included for comparison with national codes. ^c^ Mixed: Code developed by a multi-stakeholder organisation, CGR, with support for the central database by the Dutch Ministry of Health and management by an independent foundation (Stichting Transparantieregister Zorg). ^d^ Payments from medical devices companies reported as of 2016; governed by another Code (not assessed for the purpose of this paper). ^e^ Does not cover universities but covers foundations or associations affiliated to universities. ^f^ Only covers organisations that subscribe to the Code; others can join on a voluntarily basis. ^g^ Payments made from January 1, 2017 do not require prior individual consent by Healthcare Professionals and will be published on an individual basis except for transfers of value related to R&D which will remain being published on an aggregate basis. ^h^ Samples reported under different regulatory requirements. ^i^ Mandates publication on a publicly available platform, either on a company website or centralised. ^j^ Partly searchable. In the Netherlands, searching is possible by beneficiary, not by company. # Only the following payments are covered: organising and sponsorship of promotional and scientific events attended by specialists (includes travel and accommodation); support to specialist professional associations and medical institutions for scientific or professionally oriented event. ♦ Research costs excluded, except for some non-interventional research. * Cost of travel, accommodation or fees for conference participation are irrelevant (although not formally excluded) as this is not allowed in the Swedish industry code.

We have included 10 policies from nine countries (two from Latvia). Of the nine included countries, only three have legislation in place. France adopted a “Sunshine Policy” in 2011, Portugal and Latvia adopted laws mandating public disclosure in 2013 and 2014, respectively. Five other included countries (Germany, Italy, Spain, Sweden, UK) have adopted a self-regulatory approach, in most cases following the adoption of the EFPIA Disclosure Code in mid-2013. In those countries there are no government-imposed disclosure requirements and the industry has developed its own transparency measures. Exceptions to implementing the EFPIA Disclosure Code might be allowed in countries where there are national laws or regulations in place. For example, the Code of the French industry association, Les Entreprises du médicament (LEEM), states that by applying the French law, it fulfils its obligations under the EFPIA Disclosure code.^9^ In Latvia, in contrast, governmental and industry regulations coexist, with complementary roles. National regulations cover sponsorship of educational and scientific events only; industry self-regulation covers a broader range of payments to individual healthcare professionals and organisations. Finally, the Netherlands has a mixed self-regulatory system with some government involvement. Transparency provisions were implemented in 2012 by the Foundation for the Code of Pharmaceutical Advertising (CGR), a multi-stakeholder, self-regulatory organisation. Unlike other parts of the CGR code, the transparency provisions do not have a legal basis. However, the database of industry payments was set up with financial support from the Dutch Ministry of Health.

With regard to the scope of the transparency provisions, in France and Portugal the reporting requirements apply not only to the pharmaceutical industry but also to the medical device industry (and, in France, also other healthcare industries). Instead most of the voluntary codes apply only to members of the national industry trade associations, which are mainly research-based companies, although in some cases non-member companies might have chosen to abide by the rules. All the codes we analysed covered a broad range of categories of health professionals, including doctors, nurses, pharmacists, and in four countries also trainees.

With regard to the disclosure of individual data, under French, Portuguese and Latvian laws, individuals who receive payments cannot refuse disclosure. On the other hand, five of the voluntary codes include an “opt-out” clause whereby health professionals can choose not to have their name publicly reported consistent with national data protection laws (eg, the UK Data Protection Act 1998 which requires health professionals’ consent before publishing information).^10^ These payments are then published in aggregate.

Differences exist in the types of payments included and excluded from reporting. France has a threshold of €10 for individual reports, whereas in the Netherlands this threshold is €500 per company per year. While some categories of payments tend to be disclosed under most codes/policies (eg, sponsorship to attend meetings, travel and accommodation costs), the reporting of certain categories of payments varies between countries. For example, in the Netherlands sponsorships for most research and development activities do not fall within the scope of the assessed disclosure obligations, while the EFPIA Code recommends to disclose them but only on an aggregate basis. Other categories of payments fall within a grey area; for example, the reporting of fees for services in France, despite being mandated by law, is still inconsistent. Food and drink are consistently excluded from reporting under most self-regulatory schemes, as the EFPIA Code does not require these payments to be reported.

With regard to the location and searchability of the data, only the United Kingdom has a searchable central registry, from which data may also be extracted for analysis. In France, the Netherlands, and Portugal the registry is searchable but data cannot be extracted. Under the Latvian governmental regulation, the reports are published as separate PDFs on the Health Inspectorate webpage and under voluntary codes in Germany, Italy, Sweden, Spain, and Latvia, data are only available on each individual company’s website or as separate PDFs on the industry association’s webpage.

For six of the countries, data access was not deemed to be user-friendly. Only for one country, the United Kingdom, did the coders judge data access to be user-friendly. In Portugal and the Netherlands coders judged data access to be partially user-friendly. For example, data cannot be extracted and in the Netherlands it is not possible to search by company.

With regard to sanctions, all the codes that are entirely voluntary treat non-disclosure as a breach of the code of conduct and treat sanctions similarly as for other breaches. Finally, in most of the countries monitoring is passive and based on receipt of complaints.

## Discussion

### Summary of Findings

This analysis of transparency provisions implemented in nine European countries shows that although important improvements have been put in place in the past few years, significant gaps remain in disclosure requirements and their implementation. The situation differs substantially from country to country and the most striking differences are between governmental and self-regulatory approaches.

First, which health industries are included? While governmental provisions apply to all the companies operating in a country, most of the self-regulatory schemes apply only to members of a specific industry trade associations. This means that the available reports likely underestimate the true extent of pharmaceutical industry funding to healthcare professionals and organizations.

Secondly, is disclosure of individual data mandatory and complete? The majority of the voluntary codes include an “opt-out” clause, through a requirement for individual consent, that undermines the meaning of a transparency policy.

Thirdly, are reports comparable in terms of the types of payments included and excluded from reporting? Large differences exist; of particular interest is the consistent exclusion of food and drink under most self-regulatory schemes. These exclusions mean that a large proportion of industry payments to health professionals remain invisible.

Fourthly, are the data provided in a centralised and searchable database? In most of the countries data are only available as separate PDFs, thus not allowing researchers, the public and the media to access and search this information in a single analysable database.

Finally, was data access deemed to be user-friendly? Only for one of nine countries, the United Kingdom, the coders unequivocally judged the data to be user-friendly, but this was based on a brief assessment and other analysts have raised serious concerns about UK data quality and incomplete documentation.^10^

### Comparison to Similar Studies

In this detailed analysis of nine countries, we found that there are widespread inconsistencies between countries. These findings are confirmed by an overview of transparency provisions in 35 States across the European region, including some non-EU countries, published in 2017 by Mental Health Europe, a non-governmental organization.^7^ The report had a different focus compared to our study: it provided a brief overview country by country, mapping not only their “Sunshine” policies/codes but also anti-corruption laws. Also, it focused not only on transparency rules targeting the pharmaceutical industry but also on provisions requiring healthcare professionals to report payments or benefits. The study found that ten countries have either laws (Belgium, Denmark, France, Portugal, Slovakia) or regulations (Greece, Latvia, Romania, Turkey, UK) governing transparency by the industry or health professionals, and nine countries have anti-corruption legislation (Croatia, Germany, Italy, Poland, the Netherlands, Spain, Sweden, Slovenia, UK). Despite the broader focus, the report confirmed that although substantial improvements have been made in the past few years, there are still wide differences with some countries opting for legally-binding solutions and others relying on industry-self-regulations which have limitations in terms of membership coverage and the need for the recipients’ consent in order to publish the data.^7^

### Limitations

Our study has a number of limitations. First, we included only nine European countries. The decision to rely on native-language data collectors on one hand limited the number of included countries but on the other hand guaranteed greater accuracy. Second, since our focus was on research-based pharmaceutical industry payments to health professionals, we did not analyse self-regulatory codes developed by other industry sectors (eg, generic medicines, over-the-counter (OTC) medicines and medical devices). Third, we only examined rules covering disclosure by pharmaceutical companies of their payments to health professionals therefore we did not include provisions that require health professionals to disclose payments by drug manufacturers. Fourth, we analysed transparency provisions implemented by February 2017 and some policies might have already changed at the time of writing as this is a rapidly evolving policy area.

### How to Ensure Greater Transparency

Our analysis has several implications for future policy initiatives. Minimum standards for transparency reporting are needed across Europe. This would be best achieved through EU legislation that is implemented by each Member State, ensuring harmonisation among countries without existing policies. For the countries that already have legislation in place, harmonisation should only be required if current legislation is to a lower standard than what is envisioned. European legislation would also cover all the companies operating in the Member States and not only those affiliated to a specific industry trade association, as is the case with self-regulatory schemes.

Ideally, national databases should be combined in a single pan-European user-friendly searchable and downloadable database with a platform that is accessible in a variety of languages. With standardised reporting criteria, this is achievable without an undue administrative burden on companies. Moreover, the dominant pharmaceutical companies, in terms of market share, are transnational corporations whose practices are likely to be similar across different countries. All payments to healthcare professionals and organizations should be included, and all health-related industries should be required to submit reports at a minimum on an annual basis, whether or not they have provided funding to healthcare professionals or organizations. Currently, a company’s failure to report may reflect either non-compliance or a lack of payments.

The option for individuals to opt out of transparency reporting is a loophole in industry self-regulation that needs to be closed across Europe; this option has allowed only 48% of the payments to be linked to individuals in the United Kingdom in 2015,^11^ increasing to 60% of payments in 2016.^12^ Transparency provisions should not allow health professionals to decide if they want their payments disclosed; health professionals have the option to forego industry financing if they wish to avoid reporting. Another loophole is in monitoring of compliance: without monitoring, it is unclear whether companies’ reports are comprehensive or accurate. In 2016, the Japanese firm Astellas reported a discovery of omissions in its transparency reports in the United Kingdom within the context of an investigation into a marketing violation.^13^ This incidental discovery raises questions about how widespread such inaccuracies are.

Similarly to the Open Payments in the United States (the federal database created to administer the disclosure requirements of the “Sunshine Act”), the transparency reports should contain a number of variables that provide information on the products in relation to which the payment was made (eg, product codes, names and therapeutic areas) and on the recipient healthcare professional (eg, a unique identification number that link physicians’ records across program years and, possibly, to prescribing databases). This would enable researchers to further explore the association between industry payments to physicians and the cost and quality of prescribing.

Finally, additional research is needed on how differences in transparency provisions affect public information access on industry payments to health professionals. If the national differences described above reflect lack of adequate evidence on how best to ensure transparency, researchers can take advantage of the natural experiment created by these differences to resolve this question.

### Beyond Transparency

Transparency reporting is an important and necessary step but it is not a solution to undue influence from industry financing of health professionals. Such influence has been demonstrated both in relation to funding of ‘key opinion leaders’ via advisory board membership, speaker fees and contracts,^14^ and the ubiquitous everyday gifts of food and drink and invitations to events that feature in transparency databases.^15,16^ There is also some evidence of a “dose-response” in the relationship between funding and prescribing rates, with higher prescribing costs and more brand-name prescriptions among physicians in the highest quintile of industry funding,^17^ as well as increased prescribing of promoted products in relation to the numbers of meals received.^15^

To address influence, restrictions on funding are needed, not just reporting transparency. France, for example, has already taken the first steps in prohibiting sales representatives from providing physicians who are working in an outpatient setting with free samples and gifts, including gifts of food, with additional restrictions on marketing within hospitals.^18^ Additionally, since 2015, the Swedish industry code has prohibited member companies from paying for the cost of travel, accommodation or fees for participants at a conference.^19^ Such initiatives need to be extended and coupled with adequate education about pharmaceutical promotion in the medical curriculum and an increase in the funding for independent continuing professional education.

We now have an excellent evidence base, in large part due to the large-scale analyses that the US Sunshine Act has made possible, on the influence of industry funding on prescribing decisions.^15^ Funding that is known to exert undue influence has no place in provision of patient care, especially within a public healthcare system.

## Acknowledgements

We would like to thank Cristina Cabrita, Anne Chailleu (Formindep), Vita Dumpe, Juan Erviti Lopez, Maria Font, Pierre Frouard, Teresa Leonardo Alves, Brian Tielrooij, Peter Tinneman, Marc Torka for their contribution to the data collection.

Shai Mulinari received financial support from The Swedish Research Council for Health, Working Life and and Welfare (FORTE) grant no. 2016-00875. The funder had no role in study design, data collection and analysis, decision to publish, or preparation of the manuscript.

Ancel.la Santos position as Senior Policy Advisor at Health Action International is funded by the European Commission’s Consumers, Health, Agriculture and Food Executive Agency (CHAFEA), the Open Society Foundations and Camino Stiftung.

## Ethical issues

Not Applicable.

## Competing interests

Barbara Mintzes reports that she was an expert witness on behalf of plaintiffs in a Canadian class action suit concerning cardiovascular risks of a testosterone gel.

## Authors’ contributions

AF, AS, SiM, BM conceived of the study. AS coordinated the data collection. All the authors analysed the data, contributed to the writing of the paper and approved the final version.

## Authors’ affiliations

^1^Charles Perkins Centre and Faculty of Pharmacy, The University of Sydney, Camperdown, NSW, Australia. ^2^Health Action International, Amsterdam, The Netherlands. ^3^Faculty of Medicine and Institute of Clinical and Preventive Medicine, University of Latvia, Riga, Latvia. ^4^Department of Sociology, Faculty of Social Sciences, Lund University, Lund, Sweden.

## Supplementary files

Supplementary file 1. Questionnaire (Information on the Law or Code).

Supplementary file 2. List of included Codes/Laws.
