# Supplementary material for: Sunshine Policies and Murky Shadows in Europe: Disclosure of Pharmaceutical Industry Payments to Health Professionals in Nine European Countries
Source: Int J Health Policy Manag. 2018 Mar 14;7(6):504–9. doi: 10.15171/ijhpm.2018.20 (PMC6015505; doi:10.15171/ijhpm.2018.20)
Supplement: Supplementary file 1 — Questionnaire (Information on the Law or Code). [file ijhpm-7-504-s001.pdf]

Supplementary File 1. Questionnaire (Information on the Law or Code)

1. Name of person filling in this questionnaire:

2. Country:

3. Title of the law/code on disclosure: (in original language and translated to English)

4. Please list the website for the law or code on disclosure.

5. Have these transparency rules already been adopted and are they already implemented ?

- ◯ Yes already adopted and implemented (eg, companies must already abide by these disclosure rules), with a database or databases of reports already available
- ◯ Yes already adopted and implemented (definition as above), but there is not yet a database of reports available
- ◯ No, already adopted but not yet implemented

Please state date adopted; if different, date implemented, or (if not yet implemented) planned implementation date.

6. Organisation responsible (in charge of administrating the law or code):

7. Reporting period

- Annual
- Every six months
- Quarterly (4 x per year)
- Other (please specify)

8. Type of policy:

- Industry self-regulation
- Government
- Mixed - governmental and industry component (for example, disclosure rules may be established by law but monitoring and enforcement delegated to a self-regulatory body)
- Other (please specify)

9. Target industries (please select all that apply)

- [ ] pharmaceutical industry
- [ ] Medical devices industry
- [ ] Other health care organizations (please specify)

10. If you answered yes to the pharmaceutical industry, which type of companies? (select all that apply)

- [ ] Brand-name / research based
- [ ] Generic medicines
- [ ] Over-the-counter medicines
- [ ] Only applicable to member companies of an industry association
- [ ] Also applicable to non-members
- [ ] Other types of companies (please specify)

11. What types of individual people who receive payments are covered? (Please select all that apply)

- [ ] No individual people covered
- [ ] Individual medical doctors / physicians (includes both general practitioners and specialists)
- [ ] Medical students / trainees
- [ ] Pharmacists
- [ ] Nurses
- [ ] Other health professionals (please specify)

12. What types of organisations who receive payments are covered? (Please select all that apply)

- [ ] no organisations covered
- [ ] patient or consumer groups
- [ ] healthcare professional organisations / medical societies
- [ ] hospitals and/or medical centres
- [ ] universities
- [ ] Other types of organisations (please specify)

13. Are data provided separately for payments to each person or organisation, or only in aggregate? (Please check all that apply)

- [ ] Data provided on payments to named individual health professionals
- [ ] Data provided on payments to named organisations
- [ ] Data provided only in aggregate on payments to health professionals - no individuals named
- [ ] Data provided only in aggregate on payments to organisations - no specific organisations named

Any comments?

14. Is consent from the recipient required for publishing of individual payments?

- [ ] Yes
- [ ] No
- [ ] Not applicable - no individual payments published

Comments:

15. Are individual recipients provided an opportunity to review the data and submit corrections?

- [ ] Yes
- [ ] No
- [ ] Unknown - no information provided
- [ ] Not applicable - no individual payments published

Comments:

16. Scope of payments and other 'transfers of value' covered: (Please select all that apply)

Note: 'transfers of value' include gifts, products, and 'in-kind' services as well as money.

- [ ] Donations and grants
- [ ] Event sponsorship
- [ ] Travel and accomodation
- [ ] Food and drink / meals
- [ ] Gifts (non-food)
- [ ] Free medication samples
- [ ] Consultancies
- [ ] Speaker fees
- [ ] Advisory board membership
- [ ] In-kind services such as a 'diabetes nurse' for a GP practice
- [ ] Other (please specify)

17. Are any types of payments excluded from disclosure? (Please mention anything that is explicitly not covered, such as samples under the EFPIA code)

- ◯ No
- ◯ Yes

If yes, please specify:

18. Is there a financial threshold for reporting of payments (eg, at least xx euros)?

- ◯ No
- ◯ Yes

If yes, please specify the threshold:

19. Where is the database of disclosed payments located? (please select all that apply)

- ☐ On each company's website
- ☐ In a centralised database

If centralised, please specify who is managing it and list the web url for database

20. What is the format of the database or databases? (we would like to know about accessibility of data and ease of extracting information)

- ◯ Single searchable database, with an on-line search function
- ◯ Single analysable database in accessible electronic format eg, excel or equivalent
- ◯ Separate excel sheets or other analysable database format per company and/or reporting
- ◯ Period non-searchable or analysable format (eg, pdf files) Other (please specify)
- ◯

21. In your judgment, is the format user-friendly?

- ◯ Yes
- ◯ No

Main reason for your judgment

22. What is the language of the database?

- ◯ English
- ◯ Local language (if not English)
- ◯ Both local language and English

Comments:

23. Are there sanctions for non-disclosure (either financial or non-financial)?

- ◯ No
- ◯ Yes

If yes, please describe type:

24. If there are sanctions for non-disclosure, who is subject to these sanctions? (Please select all that apply)

- ◯ Companies
- ◯ Healthcare professionals or organisations
- ◯ Other (please specify)

25. Does the code include provisions for monitoring compliance? (eg, audits of companies, professionals, organisations)

No

Yes

If yes, please describe

26. Does the code include provisions to address and process complaints?

Yes

No

Comments

27. Are there time limits to the availability of the data?

No

Yes

If yes, please specify

28. Do you have any other comments? (any other aspects of the code/database that you would like to emphasise?
