# Supplementary material for: Sunshine Policies and Murky Shadows in Europe: Disclosure of Pharmaceutical Industry Payments to Health Professionals in Nine European Countries
Source: Int J Health Policy Manag. 2018 Mar 14;7(6):504–9. doi: 10.15171/ijhpm.2018.20 (PMC6015505; doi:10.15171/ijhpm.2018.20)
Supplement: Supplementary file 2 — List of included Codes/Laws. [file ijhpm-7-504-s002.pdf]

Supplementary File 2

Country	Name of the Code	Website	Organisation Responsible	Date
**Government**				
FR	Law No. 2011-2012 of 29 December 2011 on the Strengthening of Health Protection for Medicinal and Health Products	https://www.legifrance.gouv.fr/affichTexte.do?cidTexte=JORFTEXT000025053440&categorieLien=id	Ministry of Health	Law enacted December 2011; first reports made public in October 2013 (on company websites and at the website of the French National Medical Council); Central database available since June 2014
LV	Cabinet Regulation No. 378 Procedures for Advertising Medicinal Products and Procedures by Which a Medicinal<br>Product Manufacturer is Entitled to Give Free Samples of Medicinal Products to Physicians	http://vvc.gov.lv/export/sites/default/docs/LRTA/MK_Noteikumi/Cab._Reg._No._378_-_Advertising_Medicinal_Products.pdf	Health Inspectorate of the Ministry of Health of the Republic of Latvia	Law enacted November 2014; first reports made public April 2016
PL	Decreto-Lei n.º20/2013, de 14 de fevereiro article nº 159<br>Despacho n.º 12284/2014, de 06 outubro<br>Circular Informativa N.º 100/CD/100.20.200 Data 30/06/2016<br>Circular Informativa N.º 082/CD/100.20.200 Data: 30/05/2016<br>Circular Informativa N.º 012/CD/8.1.6. Data: 17/01/2014<br>Circular Informativa N.º 024/CD/8.1.6. Data: 14/02/2013<br>Decreto-Lei n.º 128/2013, de 5 de setembro<br>Decreto-Lei n.º 5/2017, de 6 de janeiro<br>Circular Informativa N.º 079/CD/100.20.200, de 30/06/2017<br>Circular Informativa N.º 003/CD/100.20.200, de 06/01/2017	http://www.infarmed.pt/documents/15786/1068535/035-G1_DL_20_2013_1ALT.pdf<br>https://placotrans.infarmed.pt/documentacao/Circulares/2550025500.pdf<br>https://placotrans.infarmed.pt/documentacao/Circulares/CircularInformativa_100_30_06_2016.pdf<br>https://placotrans.infarmed.pt/documentacao/Circulares/12012368.pdf<br>https://placotrans.infarmed.pt/documentacao/Circulares/9580300.pdf<br>https://placotrans.infarmed.pt/documentacao/Circulares/8669190.pdf<br>http://www.infarmed.pt/documents/15786/1068535/035-G2_DL_128_2013_VF.pdf<br>https://placotrans.infarmed.pt/documentacao/Circulares/Decreto-Lei_5_2017.pdf	Infarmed (National Authority for Medicines and Health Products)	Effective as of 15 February 2013

		https://placotrans.infarmed.pt/documentacao/Circulares/Circular_Informativa_079.pdf https://placotrans.infarmed.pt/documentacao/Circulares/CI_N_003-CD-06_01_2017.pdf		
**Industry Self-regulation**				
EFPIA/Europe	EFPIA Code on Disclosure of Transfers of Value from Pharmaceutical Companies to Healthcare Professionals and Healthcare Organisations	http://transparency.efpia.eu/uploads/Modules/Documents/efpia-disclosure-code-2014.pdf	European Federation of Pharmaceutical Industries and Associations	Adopted in June 2013 and amended in 2014. It mandates disclosure of transfers of value as of 30 June 2016 (for payments made in 2015)
DE	FSA-Transparency Codex ("Voluntary self-monitoring for the pharmaceutical industry")	http://www.pharma-transparency.eu/	FSA (Association of Voluntary Self-Control of the Pharmaceutical Industry)	Adopted 2014; first reports made public in 2016
ES	Code of Practice for the Pharmaceutical Industry - Farmaindustria	http://www.codigofarmaindustria.org/servlet/sarfi/elcodigo.html	Farmaindustria (National Trade Association of the Spanish based pharmaceutical industry)	Transparency provisions included in the Code in 2014; first reports made public in June 2016; revised Code entered into effect in May 2016
IT	Code of Conduct Farmindustria	http://www.farmindustria.it/index.php?option=com_jdownloads&Itemid=0&view=finish&cid=107349&catid=78	Farmindustria (association of pharmaceutical companies operating in Italy)	Adopted 2014; first reports made public June 2016

LV	Disclosure Code	http://www.siffa.lv/uploads/etika/Kodeksi%202015/Atklatibas_kodekss_140930_EN.doc	1) Association of International Research-based Pharmaceutical Manufacturers<br>2) Latvian Generic Medicines Association	Adopted: 2014 ; first reports made public April 2016
SE	Ethical rules for the pharmaceutical industry in Sweden<br>Section 3 – Disclosure of transfers of value	In Swedish:<br>http://www.lif.se/globalassets/etik/dokument/ler-svenska-pdf-20170101.pdf<br><br>In English:<br>http://www.lif.se/globalassets/etik/dokument/ler-english-pdf-20170101.pdf	LIF (Swedish Association of the Pharmaceutical Industry)	First reports made public May 2016.<br>Member companies of LIF (the industry trade group) have also disclosed: "collaborations" with patient and patient and user advocacy and interest groups since 2005; "collaborations" with professional organisations since 2007; "collaborations" with public healthcare organisations since 2011.
UK	ABPI Code of Practice for the Pharmaceutical Industry	http://www.pmcpa.org.uk/thecode/Documents/Code%20of%20Practice%202016%20.pdf	ABPI (Association of the British Pharmaceutical Industry); has established the separate<br>Prescription Medicines Code of Practice Authority to implement code.	Implemented: 30/06/2016 (for payments made in 2015)
**Mixed (predominantly self-regulatory; multi-stakeholder board)**				
NL	Code of Conduct for Pharmaceutical Advertising	http://cgr.nl/CGR.nl/media/CGR.nl/Gedragscode/20160706-Dutch_CoC_Pharmaceutical_Advertising-ENG-per-01072015.pdf	CGR (Foundation for the Code of Pharmaceutical Advertising) manages the Code. The Transparency Register Foundation<br>(Stichting Transparantieregister Zorg) was established in 2012 at the initiative of the the CGR to manage the register.	On 1 January 2012, the CGR implemented new rules on the disclosure of financial relations. A central database and webportal were set up and the first reports were published at the start of 2013.

Abbreviations: DE, Germany; ES, Spain; FR, France; IT, Italy; LV, Latvia; NL, The Netherlands; SE, Sweden; UK, United Kingdom; EFPIA, European Federation of Pharmaceutical Industries and Associations.
